# Identification of Molecular Fingerprints in Human Heat Pain Thresholds by Use of an Interactive Mixture Model R Toolbox (AdaptGauss)

**DOI:** 10.3390/ijms161025897

**Published:** 2015-10-28

**Authors:** Alfred Ultsch, Michael C. Thrun, Onno Hansen-Goos, Jörn Lötsch

**Affiliations:** 1 DataBionics Research Group, University of Marburg, Hans-Meerwein-Straße, Marburg 35032, Germany; E-Mails: ultsch@informatik.uni-marburg.de (A.U.); mthrun@informatik.uni-marburg.de (M.C.T.); 2 Institute of Clinical Pharmacology, Goethe-University, Theodor-Stern-Kai 7, Frankfurt am Main 60590, Germany; E-Mail: Hansen-Goos@med.uni-frankfurt.de; 3 Fraunhofer Institute for Molecular Biology and Applied Ecology IME, Project Group Translational Medicine and Pharmacology TMP, Theodor-Stern-Kai 7, Frankfurt am Main 60590, Germany

**Keywords:** pain, R software, bioinformatics, data modeling, molecular mechanisms

## Abstract

Biomedical data obtained during cell experiments, laboratory animal research, or human studies often display a complex distribution. Statistical identification of subgroups in research data poses an analytical challenge. Here were introduce an interactive R-based bioinformatics tool, called “AdaptGauss”. It enables a valid identification of a biologically-meaningful multimodal structure in the data by fitting a Gaussian mixture model (GMM) to the data. The interface allows a supervised selection of the number of subgroups. This enables the expectation maximization (EM) algorithm to adapt more complex GMM than usually observed with a noninteractive approach. Interactively fitting a GMM to heat pain threshold data acquired from human volunteers revealed a distribution pattern with four Gaussian modes located at temperatures of 32.3, 37.2, 41.4, and 45.4 °C. Noninteractive fitting was unable to identify a meaningful data structure. Obtained results are compatible with known activity temperatures of different TRP ion channels suggesting the mechanistic contribution of different heat sensors to the perception of thermal pain. Thus, sophisticated analysis of the modal structure of biomedical data provides a basis for the mechanistic interpretation of the observations. As it may reflect the involvement of different TRP thermosensory ion channels, the analysis provides a starting point for hypothesis-driven laboratory experiments.

## 1. Introduction

Biomedical data obtained during cell experiments, research in laboratory animals or studies in human volunteers often reveal a distribution which is more complex than a single Gaussian. This is owed to several factors that shape the numerical values of the studied parameters that may be inherent to the complexity of the studied trait, or have been introduced as treatments, or experimental conditions, or may be properties of the study subjects such as animal strain, age, sex, or a pathological condition. The influence of such concomitantly acting factors may result in a highly complex, non-normal, and often multimodal distribution of the data. As a consequence, influences of factors inducing subgroups in the data may be missed by standard statistics as these test known factors but may miss unknown influences, or prerequisites of statistical tests, such as the normality assumption, may be violated. Therefore, careful exploration of potentially complex data is required.

An example of a highly complex trait is pain, which is known to be based on a complex network of molecular nociceptive pathways [1]. This results in a complex phenotypic representation [2]. This triggered the acknowledgement of the need of detailed pain diagnosis [3,4,5]. Recent research emphasized that the complex molecular background of pain can be detected in its phenotypic representation by analyzing the multi-modal distribution of pain measures [6]. However, standard statistical software often offers only limited tools for this purpose and moreover, the availability of tools by which biologically plausible interpretations of the data structure can be obtained, is still rare.

**Figure 1:**
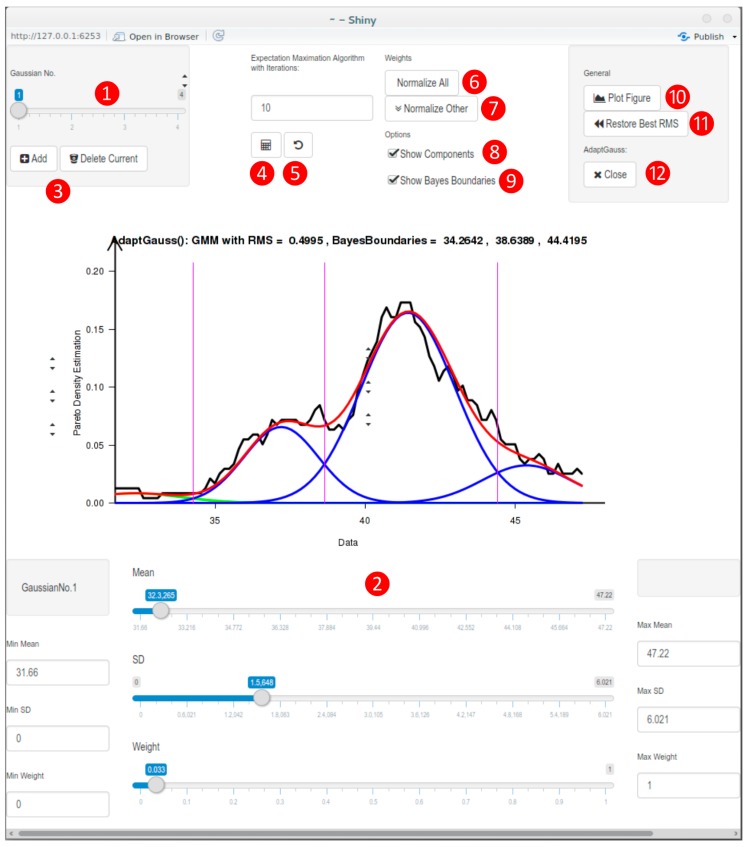
Screenshot of the interface of the AdaptGauss R tool. The following actions can be done by the user: ❶ Select the Gaussian that you intend to edit. The selected Gaussian is shown in green; ❷ Modify the parameters of the selected Gaussian; ❸ Add a new Gaussian or delete the selected one; ❹ Execute expectation maximization (EM) algorithm with displayed number of iterations; ❺ Prior to the execution of the EM algorithm the parameters of the current GMM is saved. Click here to restore these parameters; ❻ Normalize all Gaussians, so that the sum of the weights is equal to 1; ❼ Preserve the weight of the selected Gaussian, but normalize the weights of the other ones; ❽ Single Gaussians will just be shown if selected. GMM is always shown; ❾ Select to show Bayes boundaries; ❿ Plot figure for further processing; ⓫ Restore number of Gaussians and parameters of the GMM with the lowest RMS; ⓬ Close AdaptGauss. If it was started by “results = AdaptGauss(data)”, the variable “results” is now a list that contains the following elements: “Means”, “SDs”, “Weights”, “ParetoRadius”, “RMS”, and “BayesBoundaries”.

Therefore, the present work presents a bioinformatics toolbox for exploring subgroup structures that allow exploring molecular mechanisms underlying a complex structure in data acquired during pain or other biomedical research (Figure 1). It is demonstrated from the example of pain research how this interactive tool is used to identify structures in the data and identify subgroups in a human supervised manner. This provides a biologically plausible hypothesis compatible with prior knowledge from molecular pain research about the underlying pathophysiology.

## 2. Results and Discussion

Heat pain threshold data (HPT) was taken from a recent study [7,8] assessing somatosensory profiles of skin areas by applying the clinically established quantitative sensory testing (QST) battery [9,10]. The analyzed cohort consisted of 127 healthy volunteers (aged 18–36 years, mean ± standard deviation: 25 ± 3.1 years, 59 men) at which the measurements were taken at the dorsal sides of either the hand or the foot.

### 2.1. Non-Normality of Heat Pain Data

**Figure 2:**
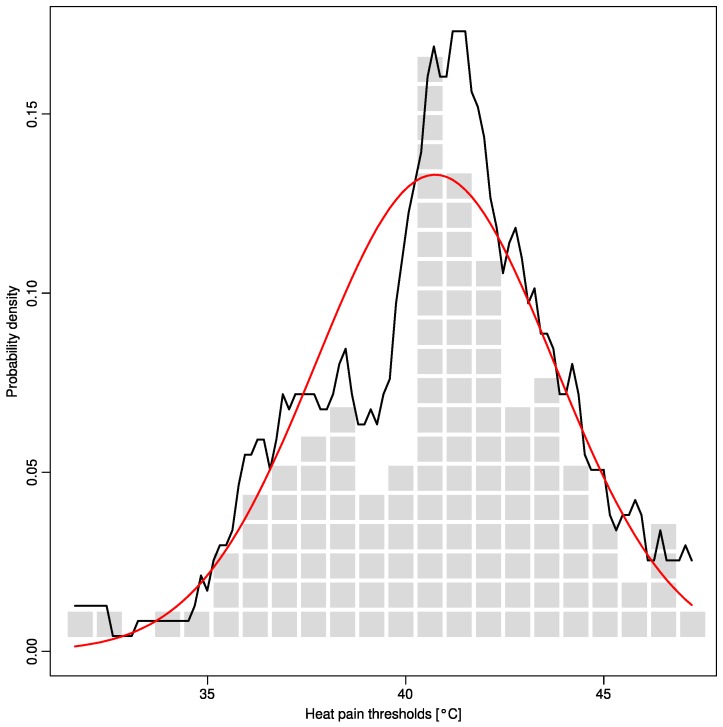
Heat pain thresholds acquired from 127 healthy volunteers (rescaled for body area and sex). The data is shown as squares. A Gaussian distribution is overlaid as a red line (mean = 40.7 °C, standard deviation = 3.0) while the Pareto Density Estimation (PDE [11] is shown as a black line. The observed distribution of the data clearly differs from the Gaussian.

Following rescaling of the data to normalize for body area and sex, a clear multimodal distribution was visually suggested (Figure 2). In particular, besides the primary mode at approximately 41 °C (64% of data), a second mode at 37 °C (20% of data) was clearly evident.

### 2.2. Multimodal Distribution of HPT Data

**Figure 3:**
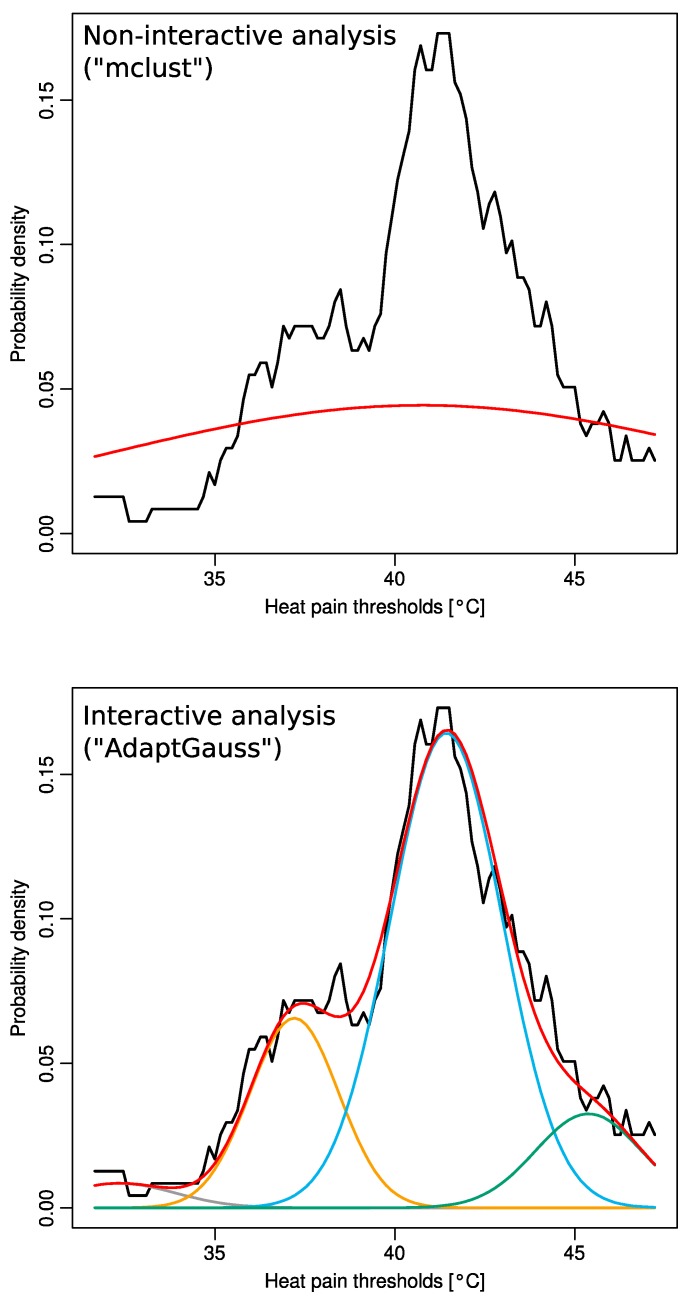
Distribution of heat pain thresholds acquired from 127 volunteers (rescaled for body area and sex). The distribution is shown as the probability density function (PDF), estimated by means of the Pareto Density Estimation (PDE [11], black line in both panels). The red line shows the Gaussian distribution of the data. Using noninteractive EM fitting of a GMM (R-library “mclust” [12]) resulted in only a single component of a Gaussian mixture (top panel, red line). By contrast, interactively adapting a GMM (“AdaptGauss”) resulted in a substantially better multimodal Gaussian mixture (brown, orange, blue and green lines) describing the overall data distribution satisfactorily (red line displaying the sum of the single Gaussians).

The data’s distribution was further explored. The histogram of the data suggested a multimodal distribution (four modes). As a standard approach to a multimodal distribution, a Gaussian mixture model (GMM) was used. This was optimized using the expectation maximization (EM) algorithm implemented in the R-package “mclust”. However, this noninteractive optimization identified only a single Gaussian with a mean of 40.7 °C and a standard deviation of 3.0, which resulted in a highly unsatisfactory fit of the data (Figure 3 top panel).

**Figure 4:**
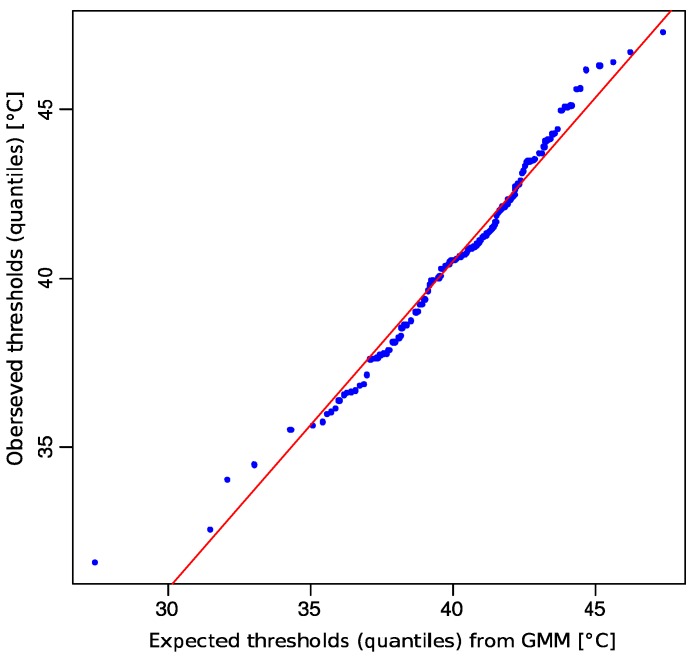
Quantile-quantile (QQ) plot comparing the observed distribution of heat pain threshold data (ordinate) with the distribution expected from the GMM (abscissa), which was fitted to the data as shown in Figure 3 bottom. The blue dots symbolize the quantiles of single observations *versus* predictions and the red line indicates identity.

A satisfactory modeling of the data could only be obtained when using an interactive approach to the multimodal distribution using the AdaptGauss toolbox presented here. Visual adaptation of four Gaussian modes such that the resulting GMM followed the data distribution closely, provided suitable start values enabling the EM algorithm to optimize a four-component mixture, which identified a GMM with means of 32.3265, 37.2006, 41.4294, and 45.3766, with associated standard deviations of 1.5648, 1.2362, 1.5624, and 1.4709, respectively, and associated weights for the Gaussians of 0.03, 0.2, 0.64, and 0.12, respectively (Figure 3 bottom panel). This model provided a good fit of the distribution of HPT data. A Kolmogorov-Smirnov test indicated this by a *p*-value of <10^−5^ in [13], which is the probability that the model does not describe the data’s distribution. The goodness of fit was also supported by visual inspection of the quantile-quantile plot (Figure 4).

Thus, the analysis showed that interactively exploring a multimodal data distribution structure is suitable to provide a satisfactory fit of pain-related measurements. Such data is often simply treated as if normally distributed, resulting in suggestions of z-transformations, as in the standard procedure for the analysis of the presently used QST test battery adherence to the instructions [9,10,14]. This disregards multimodal structures as for example shown in Figure 2 of [15]. If the rejection of normal distribution by statistical tests is acknowledged and multimodal structures are recognized, the noninteractive standard consisting of an EM based optimization of the data does not necessarily provide a satisfactory result. This owes to typical effects in the EM algorithm which is a local gradient-descend optimization algorithm aiming at maximization of the maximum likelihood. It implies that EM can only provide good solutions if started with an already rather good solution. The latter can be obtained visually when using an interactive fitting tool, as demonstrated in this report.

The results of this analysis of pain-related data with special attention to the data distribution enable the identification of potentially meaningful phenotypes suitable to derive molecular mechanism-based hypotheses. It is a known fact that thermal stimuli are recognized with distinct sensations, from lowest to highest temperatures, consisting of noxious cold, cold, warm, and noxious hot [16]. A large fluctuation of sensitivities across the skin has been observed more than 50 years ago which took two forms, *i.e.*, (i) marked changes primarily at the boundaries of the sensory fields; and (ii) “fragmentation” and “coalescence” of the fields themselves [17]. This can now be attributed to the transduction of thermal information via several different thermal sensors, of which the largest group belongs to the transient receptor potential (TRP) channels [18,19,20,21] This group of multimodal ion channels nonselectively permeable to cations, including sodium, calcium, and magnesium, includes cold (TRPM8, TRPA1), warmth (TRPV3, TRPV4), and heat sensing (TRPV1, TRPV2) members [22]. Further thermal sensors are TRPC5 being sensitive to cool temperatures of 37–25 °C [23]. Studies of the knockout of TRPV1, TRPV3, and TRPM8 genes in mice have corroborated the contributions of the respective gene products to thermal sensation (TRPV3 and TRPM8) or heat related hyperalgesia (TRPV1) [24,25,26,27,28,29,30]. Among receptors of warmth and noxious heat is TRPV1 that is activated at temperatures above 42 °C, with a *T*_50_ reported at 46.1 °C, [22], which is consistent with reported temperature thresholds of nociception [31,32] and corresponds to the HPT observations in the majority of the present subjects. By contrast, TRPV3 and TRPV4 have been suggested to be activated by innocuous heat with reported temperature threshold ranges of 30–40 and ~25–34 °C, respectively [16]. TRPV4, however, they have also been implicated in thermal and mechanical hyperalgesia [33,34]. Further heat sensors are provided by TRPV2, sensitive at high temperatures of ~52 °C [21].

Based on the above-mentioned observation of a distinction of cool and noxious cold perceptions in 11 subjects, the hypothesis has been raised that these sensations are mediated via different afferent channels [35]. The modes of the Gaussians are suggestive of the activation of distinct thermosensors. For example, TRPV4, V3, or C5 cover a temperature threshold range of 25–40 °C [16] which would accommodate the mode of the third Gaussian at 41.4 °C. By contrast, TRPV1 is activated at temperatures above 42 °C with a *T*_50_ of 46.1 °C which would accommodate the mode of the forth Gaussian at 45.4 °C. In addition, the second mode at 37.2 °C falls within the activation temperature range of TRPV3 whereas the first Gaussian, because of its weight of only 0.03, might not merit further regard. Molecular research based on this data observation, in particular Gaussians 2–4, would assess the possibility of relative differences in local expression or activities of these TRP channels. This provides candidate genes for the assessment of a possible genetic cause of the split of the subjects into distinct subgroups of heat pain thresholds.

The present results emphasize the multimodal distribution of thermal pain thresholds. This has been often neglected [15]. It similarly applies to cold pain thresholds, as shown previously based on a similar approach as that presently employed [6]. Cold sensation is also transmitted via several different sensors. For example, TRPM8 is gated by menthol, icilin, and innocuous cold (18–25 °C) [36]. It seems to be necessary for both, proper cold detection and cold-induced analgesia, as shown in TRPM8 knockout mice [28]. Its *T*_50_, *i.e.*, the temperature at which half of the expressed channels are activated, has been identified at 18.0 °C [22]. A role of TRPA1, which is usually also regarded as a noxious cold sensor [37], has been differently concluded from experiments in TRPA1 knockout mice [38,39]. This suggested, in the absence of TRPA1, either a preservation of the sensitivity to cold [38] or a significantly reduced responsiveness to noxious cold [39]. TRPPA1 starts to sense cold at a temperature of 17 °C [40]. As shown previously in an analysis similar to the present one, the temperature ranges of TRPM8 and A1 are suitable to have caused the occurrence of distinct Gaussians in human cold pain thresholds with modes at 24 and 15 °C observed previously [6].

A further example of the suitability of an interactive GMM for providing a biologically-plausible hypothesis about molecular data is a recent identification of methadone induced DNA hypermethylation [41]. Following three days of incubation of MCF7 cells with several known or potential modulators of DNA methylation including the test drug methadone, global DNA-methylation, quantified at LINE-1 CpG islands [42], showed a large variability across all treatments. Based on an interactive GMM distribution analysis, the largest proportion of methadone-treated samples was found in the Gaussian comprised the samples with the highest degree of DNA methylation. This proved to be statistically significant in a Fisher’s exact test; hence, an opioid-induced DNA hypermethylation could be made possible by allowing to take a “bird’s eye” view on pooled biomedical data that enabled identifying a structure that well reflected the different experimental conditions to which the cell lines had been subjected [41]. Note that those results were not found with current standard data analysis approaches; hence, the GMM approach was able to provide a basis that encourages further experimental exploration of epigenetic opioid effects rather than a final result.

## 3. Methods

### 3.1. Heat Pain Threshold Data (HPT)

The data was taken from a recent study [7,8] assessing somatosensory profiles of skin areas by applying the clinically established quantitative sensory testing (QST) battery [9,10]. To the originally enrolled 110 healthy subjects further 17 were added from a continuing effort at sensory profiling. All assessments followed the Declaration of Helsinki and were approved by the Ethics Committee of the Goethe-University, Frankfurt am Main, Germany, and subjects had provided informed written consent. Exclusion criteria were a current clinical condition, excluded by medical history and physical examination, and intake of drugs during the previous week except for oral contraceptives and hormone substitution therapies.

This QST battery includes thermal and mechanical stimuli grouped into seven tests of sensory perception and pain, which provided a total of 13 different QST parameters as described previously [7,8]. For the present report, pain thresholds to noxious heat were selected. Thermal pain thresholds were assessed on a 9 cm^2^ skin area using a 3 × 3 cm^2^ thermode (TSA 2001-II, MEDOC, Ramat Yishai, Israel). Heat pain thresholds (HPT) were measured by increasing the thermode’s temperature by 1 °C/s, starting at 32 °C, until the subject indicated pain, which triggered the reversal of the temperature ramp back to baseline. The HPT was defined as the mean of three measurement repetitions. During testing the room temperature was kept at 20–25 °C. All measurements were taken by trained investigators fully adhering to published instructions [9,10,14].

The cohort consisted of 127 healthy volunteers (aged 18–36 years, mean ± standard deviation: 25 ± 3.1 years, 59 men) at which measurements had been either at the dorsal side of the hand (*n* = 72 subjects) or at the dorsal side of the foot (*n* = 55) of the left (*n* = 65) or the right (*n* = 62) body side (χ^2^ test body area *versus* side: χ^2^ = 0.234, d*f* = 1, *p* = 0.6285). The body side had no statistically significant effects on the HPT (Wilcoxon rank sum test [43]: *W* = 2276, *p* = 0.2089). However, as expected from previous reports about body area differences in thermal thresholds [44,45] including neighboring areas such as the thenar eminence, fingers, and forearm [46], data differed statistically significantly with respect to the area from which it had been acquired (Wilcoxon rank sum test: *W* = 2459, *p* = 0.0199). Therefore, body area as a potential confounder was removed by z-transforming the values for the foot area and rescaling them such that their mean and variance became identical to that of the hand area. In addition, HPT differed also among sexes (Wilcoxon rank sum test: *W* = 2701, *p* = 0.000787), which again agrees with prior evidence, namely about sex differences in pain perception (for reviews, see [47,48,49]). Therefore, HPT data was also corrected to standardize for sex.

### 3.2. Beyond Normal Distribution: Gaussian Mixture Models

If the distribution of data is more complex than one single normal (Gaussian) distribution, the underlying process which produces the data may operate in different states, which have their own factors to influence the measurements. The usage of so called mixture models is a standard statistical tool for such applications [50,51]. Gaussian mixture models represent the present of subclasses within a complete data set using a weighted sum of single components. Each component (mode) consists of a single Gaussian parameterized with mean and standard deviation [52] (for further details, see also [53]). Specifically, a mixture of Gaussian distributions (Gaussian mixture model (GMM)) is a weighted sum of *M* component Gaussian densities as given by the equation:

(1) $$p\left(x\right)=\;\sum_{i=1}^{M}w_{i}N\left(x\text{|}m_{i},s_{i}\right)=\sum_{i=1}^{M}w_{i}\cdot \frac{1}{\sqrt{2\pi s_{i}}}\cdot e^{-\frac{{\left(x-m_{i}\right)}^{2}}{2s_{i}^{2}}}$$

where *N*(*x|m_i_*, *s_i_)* denotes Gaussian probability densities (component, mode) with means *m_i_* and standard deviations, *s_i_*. The *w_i_* are the mixture weights indicating the relative contribution of each component Gaussian to the overall distribution which add up to a value of one. *M* denotes the number of components in the mixture. Usually the parameters of the GMM, including the number of components M, are optimized using the expectation maximization (EM) algorithm [54]. The R program “mclust” [12], for example, provides such an optimization.

However, as expectation maximization (EM) represents a gradient descending algorithm for the Maximum Likelihood function, it may lead only to good solutions if a good initial solution is given. Even the EM may converge to a local maximum. To address this problem of EM, an interactive tool for a visually guided selection of optimal parameters for EM, called “AdaptGauss” has been realized. The quality of the resulting GMM can be visually addressed by a QQ-plot (Figure 4) and statistical testing for the identity of the data and the GMM distribution function. The popular χ^2^ test is an example for such a quality assessment.

Within the framework of GMM there is no need to provide *a priori* the sub-population (class, mode, component) to which an individual observation belongs. With the GMM the Bayes’ Theorem can be used [55]. Using the Bayes theorem, the probability that an individual observation belongs to mode *i*, can be calculated (posterior probability). The decision boundaries between four modes are the lines of minimal risk for misclassification. They are located at the intersection of the component’s distributions (see the magenta lines in Figure 1).

### 3.3. Implementation of a Visualization Guided GMM Tool: AdaptGauss

An interactive tool to optimize the parameters of a GMM, called “AdaptGauss”, was realized using the freely-available R software package (version 3.2.2 for Windows/version 3.2.1 for Linux; [56]). The newly devolved R library “AdaptGauss” is freely available at [57]. For the graphical interface the open source web application framework Shiny for R was used. AdaptGauss works on univariate data. After running “AdaptGauss()” the interactive interface shown in Figure 1 appears. A kernel-based density estimation, called Pareto density estimation (PDE) [11], is calculated and displayed as a black line. The PDE serves to estimate the probability density function (PDF) of the data and consists of a kernel density estimator representing the relative likelihood of a given continuous random variable taking on specific values. PDE has been shown to be particularly suitable for the discovery of structures in continuous data hinting at the presence of distinct groups of data and particularly suitable for the discovery of mixtures of Gaussians [11]. Specifically, in the PDE the data is slivered in kernels with a specific width; this width, and therefore the number of kernels, depends on the data. In AdaptGauss the data’s PDE can be approximated by a Gaussian Mixture Model (GMM), which is the weighted sum of several Gaussians. The user has the option to remove mixture components of the GMM, add new ones, or change the parameter of the individual Gaussians. The EM algorithm can also be used to optimize the used parameters. The similarity of the GMM and the PDF is measured via the root mean square (RMS):

(2) $$RMS=\sqrt{\frac{1}{N}\sum_{i=1}^{N}{\left[PD\left(x_{i}\right)-GMM\left(x_{i}\right)\right]}^{2}}\;,\;with\;N:number\;of\;kernels$$

The RMS that is displayed in AdaptGauss is normalized with respect to the mean and standard deviation of the data. The limits between the different Gaussian states are defined by Bayes decision boundaries [58] (magenta line in Figure 1). Thus, the probability a data point being assigned to a specific Gaussian is calculated by an application of the Bayes’ Theorem [55]. In comparison to the maximum likelihood criterion used as the standard in EM optimization, RMS often seems to produce more meaningful results. The interactive tool allows to visually adjust the fit, *i.e.*, the numerical values were chosen interactively with the RMS as the criterion and the resulting model was tested with respect of the goodness of data fit using the quantile-quantile plot approach and the Kolmogorov-Smirnov test [13] assessing the probability that the obtained model did not describe the observed data distribution.

## 4. Conclusions

In the present report, a novel tool is proposed for the Gaussian mixture modeling (GMM) analysis of biomedical data. It can be applied to empirical data which has a multimodal structure. The implementation of an interactive interface allowed identifying suitable start values for the expectation maximization (EM) algorithm. This resulted in a successful fit of a four component GMM that described the sample data consisting of heat pain thresholds much more plausible than a one component model. The results provide a basis for the mechanistic interpretation of the HPT observations was reflecting the involvement of different TRP thermosensory ion channels which can serve as a start point for hypothesis-driven laboratory experiments.

The present analysis of heat pain thresholds suggests that the underlying molecular mechanisms could be assessable more distinctly when the structural patterns and distributions of the data are taken into account rather than applying standard analyses. Indeed, as with cold pain thresholds, heat pain sensitivity seems complex, which is usually not regarded, as standard analytical approaches assume unimodal normal or normalized distributions. Finally, it can be mentioned that the present approach is not restricted to pain; findings presented here illustrate the likely suitability of advanced data analysis, with special regard to data distribution, for identifying potentially meaningful structures interpretable as molecular fingerprints and, thus, providing biologically-plausible hypotheses about molecular data.
